# DNA Microarray-Based Screening and Characterization of Traditional Chinese Medicine

**DOI:** 10.3390/microarrays6010004

**Published:** 2017-01-30

**Authors:** Ryoiti Kiyama

**Affiliations:** Biomedical Research Institute, National Institute of Advanced Industrial Science and Technology (AIST), 1-1-1 Higashi, Tsukuba 305-8566, Ibaraki, Japan; kiyama.r@aist.go.jp; Tel.: +81-29-861-6189

**Keywords:** DNA microarray, traditional Chinese medicine, signaling pathway, estrogen, food chemicals

## Abstract

The application of DNA microarray assay (DMA) has entered a new era owing to recent innovations in omics technologies. This review summarizes recent applications of DMA-based gene expression profiling by focusing on the screening and characterization of traditional Chinese medicine. First, herbs, mushrooms, and dietary plants analyzed by DMA along with their effective components and their biological/physiological effects are summarized and discussed by examining their comprehensive list and a list of representative effective chemicals. Second, the mechanisms of action of traditional Chinese medicine are summarized by examining the genes and pathways responsible for the action, the cell functions involved in the action, and the activities found by DMA (silent estrogens). Third, applications of DMA for traditional Chinese medicine are discussed by examining reported examples and new protocols for its use in quality control. Further innovations in the signaling pathway-based evaluation of beneficial effects and the assessment of potential risks of traditional Chinese medicine are expected, just as are observed in other closely related fields, such as the therapeutic, environmental, nutritional, and pharmacological fields.

## 1. Introduction

Herbal medicine is an important part of the medical practices in traditional Chinese medicine (TCM), and consists of a variety of plant species [1,2]. While the effectiveness of herbal medicine has sometimes been considered doubtful [3,4], this could be due in part to the difficulty of controlling the quality of herbs and the amounts of their effective components. Thus, various methods have been used to confirm their efficacy and identify the effective components [5]. Modern technologies, such as high-performance liquid chromatography (HPLC) and quantitative reverse-transcription polymerase chain reaction (qRT-PCR), have been continually developed and utilized to replace conventional technologies for the comprehensive and cost-effective quality control of herbal medicine [6]. Although recent progress in the human and other genome projects and the development of a variety of omics technologies—such as genomics and transcriptomics—have contributed to the identification and utilization of effective components and quality control/authentication of medicinal plants [7,8,9,10,11], we are still searching for the best strategy for the maximum utilization of herbal medicine. This review focuses on DNA microarray-based gene expression profiling, a key technology in transcriptomics, and shows how researchers used this technology for the screening and characterization of TCM. In particular, cross-examination of the data on TCMs, and their constituent herbs, mushrooms, and dietary plants, has become quite important to evaluate the knowledge accumulated and to assess the advantages/disadvantages of TCM and its effective applications.

TCM includes practices such as acupuncture, moxibustion, Chinese herbal medicine, tui na, dietary therapy, tai chi, and qi gong, which is rooted in the ancient philosophy of Taoism and is more than 2500 years old [12]. The original TCM has been further developed and modified to adapt it to people of various nationalities and genetic backgrounds (giving rise to different types of health problems, dietary and nutritional customs/practices, and ways of thinking and beliefs about medicine), based on variations in the types of herbs and their ingredients in countries such as Japan and Korea, where Kampo and traditional Korean medicine (TKM), respectively, were developed. Therefore, herbal medicine includes a number of plant species and ways of processing them. Furthermore, herbal medicine is often used with other constituents such as mushrooms and dietary plants, and thus their extracts and effective chemicals are also discussed here.

DNA microarrays are a type of biotechnological device used to detect alterations in genomic DNA and mRNA and to monitor genes and their expressions associated with various functions; thus, they have been widely used in basic research and industrial research/development (reviewed by Kiyama & Zhu [13]). DNA microarray assay (DMA) has been used to screen and characterize useful materials among mixtures of chemicals and extracts of natural resources including plants. DMA has advantages and disadvantages compared with other technologies. It has been used as a diagnostic device, such as for the genotyping of drug-metabolizing genes and predicting the metastatic risk of breast cancer, which is attributable to its unique characteristic of providing sufficient complexity to differentiate the variations needed for diagnosis and the reliability needed to predict genotypes/gene expression profiles accurately.

## 2. Herbal Medicine, Effective Chemicals, and Their Effects

### 2.1. Herbs, Mushrooms, and Dietary Plants Analyzed by DNA Microarray Assays

A number of herbs, mushrooms, and dietary plants, including those used as TCM in China, Korea (TKM), and Japan (Kampo), have been analyzed by DMA (Table 1).

Extracts from herbs have been analyzed by DMA, including the following: extracts of the whole, or parts such as flower and leaves, of alkanet root, American ginseng, barbed skullcap, beach vitex, beth root, black cohosh, cancer bush, Chinese figwort, boneset, dong quai, field horsetail, greater celandine, kava, leigongteng, moutan, mum, orchid, pink lapacho, purple coneflower, salai, St. John’s wort, turmeric, and wild yam. Meanwhile, the extracts of root, radix or rhizome were made from Chinese goldthread, Chinese peony, cistanche, danshen, goldthread, huang-qi, kudzu, and sheng-di-huang; the extracts of seeds were from lotus; or the extracts enriched in essential oil were from lovage and turmeric.

The extracts from mushrooms were also analyzed after extraction of the whole or the fruiting body, such as that from buna-shimeji, caterpillar fungus, common mushroom, himematsutake, hiratake, lingzhi, lumpy bracket, maitake, and turkey tail; after extraction of the mycelium, such as that from caterpillar fungus, himematsutake, lingzhi, lumpy bracket, maitake, shiitake, and turkey tail; or after extraction of polysaccharides from lingzhi or triterpenes from hiratake, hoelen, and lingzhi.

Extracts were made from mixtures of TCM, such as the following: Danggui Buxue Tang, Guanxin No. 2 decoction, Huang-Lian-Jie-Du decoction, ISF-1, Kangxianling, PC-SPES, Pulsatillae decoction, Qingfei Xiaoyan Wan, Qinggan Huoxuefang, S/B remedy, Si-Wu-Tang, VI-28, Xiaoqinglong decoction, Xuefu Zhuyu decoction, and Zeng Sheng Ping (TCM); Chunggan, SH21B, and Youkongdan (TKM); or Boiogito, Bofutsushosan, Orengedokuto, Hochu-ekki-to, Inchin-ko-to, Juzen-taiho-to, Kososan, Saireito, Toki-shakuyaku-san, and Toki-to (Kampo).

Extracts were made from other dietary plants (including vegetables, fruit, and cereals), such as bilberry, bitter gourd, buckwheat, carob, Chinese mahogany, Chungkookjang (fermented soybean), ginger, gromwell, kothala himbutu, Marie Ménard apple, and tarragon, or more common food materials such as apple, black raspberry, blueberry, broccoli, citrus, clove, garlic, ginkgo, grape, grapefruit, green tea, kiwi fruit, lychee, nectarine, oil palm, olive, peach, persimmon, pistachio, soybean, and sweetcorn.

### 2.2. Effective Chemicals Characterized by DNA Microarray Assays

After characterization of the extracts of herbs, mushrooms, or dietary plants, effective chemicals have been enriched or, in some cases, purified. They were then analyzed by DMA in order to identify the functions of interest or the signaling pathways involved (Table 2).

Pure chemicals analyzed by DMA are as follows: actein (a triterpene glycoside), aculeatin (a coumarin), baicalin (a flavone glycoside), berberine (an isoquinoline alkaloid), biochanin A, boswellic acid (a triterpene), brefeldin A (a lactone antibiotic), celastrol (a quinine methide triterpene), chelidonine (a tertiary alkaloid), curcumin (a diarylheptanoid), deoxycholic acid (a steroid acid), 3,3′-diindolylmethane (an indole-3-carbinol derivative), emodin (an anthraquinone derivative), ergosterol peroxide (a steroid derivative), genistein (an isoflavone), ginsenosides F1/Rb1/Re/Rg1/Rg3/Rh1 (steroid glycosides/triterpene saponins), glycyrrhizin (a pentacyclic triterpenoid), grifolin (a farnesylphenol/sesquiterpenoid), (−)-hydroxycitric acid (a derivative of citric acid), β-hydroxyisovalerylshikonin (a naphthoquinone derivative), jasminoidin (a geniposide), ligustrazine (a tetrapyrazine), lycopene (a carotene), myricetin (a flavonol), obovatol (a biphenolic), paeoniflorin (a monoterpene glycoside), paeonol (an acetophenone derivative), 1,2,3,4,6-penta-*O*-galloyl-β-d-glucose (PGG), plumbagin (a naphthoquinone derivative), polysaccharide-K (Krestin) (a protein-bound polysaccharide), quercetin (a flavonol), resveratrol (a stilbenoid), saffron (a carotenoid), salvianolic acid B (a tanshinol/caffeic acid), sesamin/episesamin/sesamolin (lignans), siallyl trisulfide (an organosulfur compound), sparstolonin B (a xanthone/isocoumarin), sulforaphane (an isothiocyanate), tanshinone IIA (a phenanthrene-quinone derivative), 2,4,3′,5′-tetramethoxystilbene (a phenylpropanoid), and triptolide (a diterpenoid epoxide).

On the other hand, mixtures of chemicals analyzed by DMA are: grape antioxidant dietary fiber (roughage/dietary fiber), oil palm phenolics, phytosterol mixtures (mixtures of steroid compounds), plant phospholipid/lipid conjugates, polysaccharides, and polyunsaturated fatty acids (PUFAs).

### 2.3. Biological/Physiological Effects Identified by DNA Microarray Assays

Biological/physiological effects and medicinal efficacy have been examined by DMA. To achieve this, a variety of assay systems have been used (Table 1), such as with different species (humans; animals, such as the chicken, dog, guinea pig, mouse, and rat; or microbes such as yeast and bacteria), tissues (brain, intestine, kidney, liver, lung, muscle, peripheral blood, or spleen) and cells (adenocarcinoma cells, alveolar epithelial cells, breast carcinoma cells, colon carcinoma cells, colorectal cancer cells, dendritic cells, dermal fibroblasts, endothelial cells, gingival fibroblasts, head and neck squamous cell carcinoma (HNSCC) cells, hepatoma cells, human umbilical vein endothelial cells (HUVECs), keratinocytes, lens tumor cells, leukemia cells, macrophages, neuroglial cells, oral squamous cell carcinoma cells, osteosarcoma cells, pancreatic cancer cells, peripheral blood mononuclear cells (PBMCs), preadipoctyes, prostate cancer cells, rat intestinal microvascular endothelial cells (RIMECs), retinal cells, or skin fibroblasts); the assays examining the statuses in vitro (using cultured normal or cancer cells, or yeast or bacterial cells, such as A549, BxPc-3, Caco-2, colo 205, DU145, ECV304, H9c2, HaCaT, HepG2, HCT-116, H4IIE, HL-60, Hs27, HT-29, J774.1, LT97, MCF-7, MDA-MB-231, MG-63, MonoMac6, NG108-15, PC-3, RAW 264.7, THP-1, 3T3-L1, UM1, UMSCC1, and YPK-1/4 cells) or in vivo (using tissues or cells from animals, or from healthy or diseased individuals); and DNA microarray platforms and assay protocols, such as those from ABioscience, Affymetrix, Agilent Technologies, Applied Biosystems, Clontech, GE Healthcare, Illumina, Mitsubishi Rayon, SuperArray, and Takara, or customized ones (see Section 3).

The biological/physiological effects analyzed are as follows: the functions/effects examined are angiogenesis modulation, anti-adipogenesis, anti-atherosclerosis/anti-arteriosclerosis, antibiotic effect, anti-carcinogenesis/anti-metastasis, antidepressant effect, anti-diabetic/anti-obesity effect, anti-endotoxin action, anti-fibrotic effect, anti-inflammation/anti-remodeling, anti-mitotic effect, apoptosis, cardioprotection, cell proliferation/differentiation, chemoprevention, cytotoxicity, DNA damage prevention, hepatotoxicity, immune response, inflammatory response, leukocyte function, neuromodulation/neuroprotection, skin aging prevention, stress response, and wound healing. The assays revealed the receptor-related signaling, such as by aryl hydrocarbon receptor (AhR), insulin receptor, peroxisome proliferator-activated receptor (PPAR), and Toll-like receptor (TLR), or hormone/growth-factor-related signaling, such as estrogen signaling, IFα/IFβ signaling, insulin-like growth factor 1 (IGF-1) signaling, and tumor necrosis factor α (TNF-α)/tumor growth factor β1 (TGF-β1) signaling, or signal-mediator-related signaling, such as caspase-3, extracellular signal-regulated kinase (ERK), mitogen-activated protein kinase (MAPK), nuclear factor κ-light-chain-enhancer of activated B cells (NF-κB), p53, and Wnt, or diseases/disorders, such as Alzheimer’s disease, circulation disorders, gynecological diseases, lipid metabolism disorders, obstructive lung disease, and Parkinson’s disease.

Meanwhile, the functions/effects identified by the analysis of pure chemicals (summarized in Table 2) are as follows: anti-carcinogenesis (actein, berberine, biochanin A, celastrol, chelidonine, genistein, ginsenoside Rg3, grape antioxidant dietary fiber, grifolin, lycopene, paeoniflorin, PGG, plant phospholipid/lipid conjugate, plumbagin, polysaccharide-K (Krestin), polysaccharides, PUFAs, quercetin and salvianolic acid B); anti-atherosclerosis (brefeldin A and phytosterol mixture); anti-inflammation (ergosterol peroxide, glycyrrhizin, and paeonol/paeoniflorin/albiflorin); immune response (celastrol, obovatol, and triptolide); anti-diabetic/anti-obesity response ((−)-hydroxycitric acid and ginsenoside Re); anti-infectious (berberine); apoptosis (curcumin, emodin, β-hydroxyisovalerylshikonin, tanshinone IIA, and 2,4,3′,5′-tetramethoxystilbene); anti-oxidative response (curcumin); adipogenesis/angiogenesis (aculeatin and sparstolonin B); cardio-, neuro-, or vasoprotection (ligustrazine, oil palm phenolics, resveratrol, and saffron); cell proliferation (PUFAs); chemoprevention (boswellic acid, myricetin, and sulforaphane); estrogen signaling (3,3′-diindolylmethane, ginsenosides F1/Rb1/Rg1/Rh1, and glycyrrhizin); ischemic stroke (baicalin/deoxycholic acid/jasminoidin); hypoxia (paeonol); life-span extension (curcumin and diallyl trisulfide); lipid metabolism (sesamin/episesamin/sesamolin); and Rho/ROCK (Rho-associated protein kinase) signaling (tanshinone IIA).

## 3. Mechanisms of Action by Traditional Chinese Medicine

DNA microarrays for gene expression analysis can be categorized into two types, global and focused DNA microarrays, based on their application [13,182]. Global DNA microarrays contain thousands to hundreds of thousands of probes representing some or all of the cDNA, expressed sequence tags (ESTs), and various types of expression marker, such as those for the estimation of mRNA copy numbers within cells. Meanwhile, focused DNA microarrays contain a few dozen to thousands of probes designed for specific purposes, such as the study of tissue/cell-type specificity, functional specificity, and expression profiling. Focused DNA microarrays are sometimes more appropriate for the study of the mechanisms of action when the action is known, such as in the case of comparative risk assessment of chemicals and the prediction of cancer metastatic risks.

The genes used in customized or focused DNA microarrays for basic research and the development of applications of TCM are as follows: sets of human apoptosis genes [38], 96 cancer-related genes [24], 225 genes related to chemotaxis/antigen processing/cell signaling/apoptosis/immune-related functions [28], mouse immunology-related genes [31], and 100 genes related to cardiac diseases, apoptosis, cell cycle/proliferation, cytokine/inflammatory, and antioxidation [43], for the study of herbs; genes related to growth factors/receptors, extracellular matrix components, proteases/inhibitors, and oncogenes/tumor suppressors [65], cell cycle-related genes [62,63], 172 human estrogen-responsive genes [53], and human pancreatic adenocarcinoma genes [64], for the study of mushrooms; sets of 3000 prostate-derived genes [78] and 1536 brain genes [72], for the study of TCM/TKM/Kampo; sets of 172 human estrogen-responsive genes [127], human drug metabolism-related genes [116], 209 inflammation/immune responsive genes [109], 2304 genes expressed in Caco-2 cells [115], 204 genes related to the immune response [121], and human apoptosis-related genes [130], for the study of dietary plants.

### 3.1. Genes and Pathways Responsible for the Action

The signaling pathways analyzed by DMA are as follows (see Kiyama & Zhu [13]; Kiyama et al. [183]): MAPK (such as G protein–coupled receptor (GPCR)/MAPK, MAPK/c-Jun N-terminal kinase (JNK), and NF-κB/MAPK/ERK) and other (such as angiogenesis, ErbB/human epidermal growth factor receptor (HER), nuclear receptor, and ubiquitin/proteasome) signaling pathways, or apoptosis pathways (such as those for death receptor, infectious response, and p53-dependent apoptosis), autophagy pathways (such as those for phosphatidylinositol-3-kinase (PI3K)/Akt/mTOR signaling and starvation stress response), cell cycle/DNA damage pathways (such as G1/S checkpoint and G2/M DNA damage checkpoint signaling pathways), cellular metabolism pathways (such as AMP-activated protein kinase (AMPK) and insulin receptor signaling pathways), chromatin/epigenetic regulation pathways (such as those for DNA methylation, heterochromatin, and histone modification), cytoskeletal regulation and adhesion pathways (such as those related to actin, adherens junction, and microtubule dynamics), development and differentiation pathways (such as hedgehog, Notch, TGF-β, and Wnt/β-catenin signaling pathways), immunology and inflammation pathways (such as those for B-cell receptor signaling, cytokine receptor signaling, inflammatory response, rheumatoid arthritis, T-cell activation, and TLR-induced immune response), neuroscience pathways (such as Alzheimer’s disease- and Parkinson’s disease-related signaling pathways) and translational control pathways (such as eIF2, eIF4/P70S6K, and mTOR signaling pathways). 

**Figure 1:**
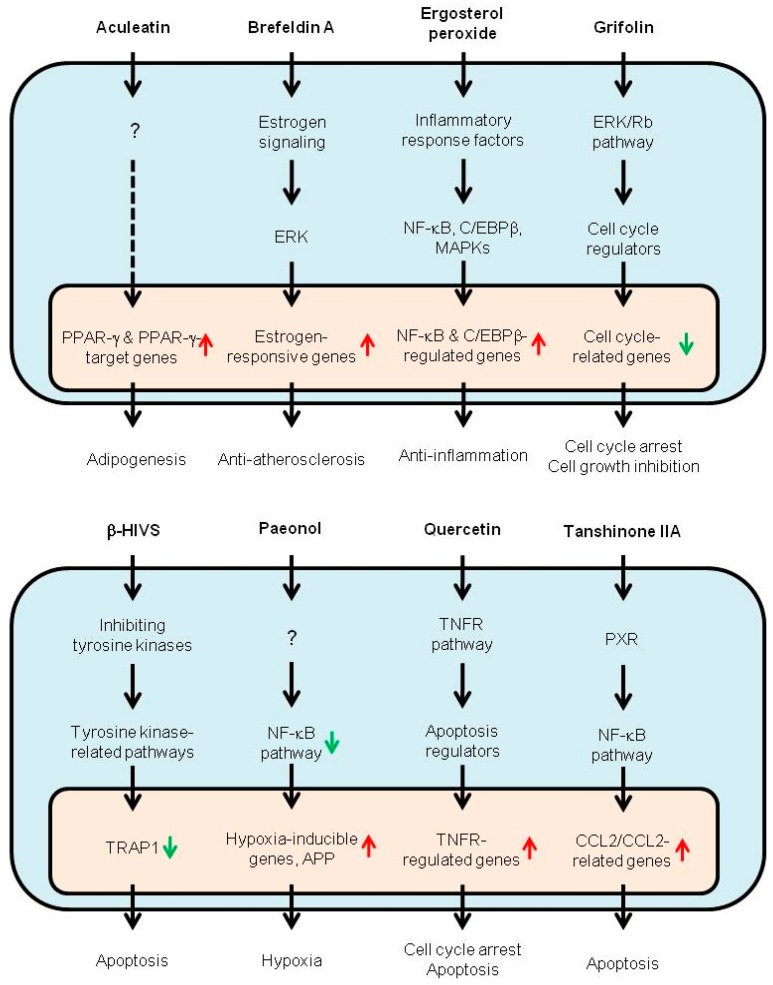
Summary of actions and their mechanisms by the chemicals related to traditional Chinese medicine. The mechanisms of action by the chemicals originally identified or isolated from medicinal herbs, mushrooms and dietary plants (aculeatin, brefeldin A, ergosterol peroxide, grifolin, β-hydroxyisovalerylshikonin, paeonol, quercetin and tanshinone IIA) within the cytosol (blue area) or the nucleus (yellow area) are summarized. APP: amyloid precursor protein; CCL2: chemokine (C-C motif) ligand 2; ERK: extracellular-signal-regulated kinase; β-HIVS: β-hydroxyisovalerylshikonin; MAPK: mitogen-activated protein kinase; PPAR-γ: peroxisome proliferator-activated receptor γ; PXR: pregnane X receptor; Rb: retinoblastoma protein; TNFR: tumor necrosis factor receptor; and TRAP1: tumor necrosis factor receptor-associated protein 1.

Since genes and pathways responsible for the action of TCM are related to various cell functions, it is almost impossible to understand the mechanisms of action just by studying the mixture of chemicals. There are cases in which effective chemicals (such as those shown in Table 2) were analyzed in order to understand specific mechanisms, such as Bax signaling/apoptosis (2,4,3′,5′-tetramethoxystilbene), ERK signaling/anti-atherosclerosis (brefeldin A), ERK signaling/anti-carcinogenesis (grifolin), estrogen signaling (ginsenosides F1/Rb1/Rg1/Rh1 and glycyrrhizin), estrogen signaling/carcinogenesis (3,3′-diindolylmethane), HSP70 (a 70 kilodalton heat shock protein) signaling/anti-carcinogenesis (paeoniflorin), NF-κB signaling/anti-carcinogenesis (quercetin), NF-κB signaling/anti-inflammation (ergosterol peroxide), NF-κB signaling/apoptosis (tanshinone IIA), NF-κB signaling/hypoxia (paeonol), Nrf2-antioxidant response element (ARE) signaling/chemoprevention (myricetin), PI3K-Akt signaling/chemoprevention (sulforaphane), PPAR-γ signaling/adipogenesis (aculeatin), reactive oxygen species (ROS) signaling/apoptosis (β-hydroxyisovalerylshikonin), Rho/ROCK signaling/cell migration (tanshinone IIA), skn-1 signaling/life-span extension (diallyl trisulfide), and tumor necrosis factor receptor 1 (TNFR1)-IGF-1R signaling/apoptosis (emodin). These signaling pathways are summarized in Figure 1.

### 3.2. Cell Functions Involved in the Action

The major cell functions analyzed by DMA for TCM include: adipogenesis, anti-atherosclerosis, anti-carcinogenesis, anti-inflammation, apoptosis, carcinogenesis, chemoprevention, hypoxia, and life-span extension (Table 2; Figure 1).

Adipogenesis is a cellular differentiation process in whichpreadipocytes are transformed into differentiated adipocyte cells, and involves features such as morphological change, growth arrest, lipogenic gene expression, and the production of hormones and growth factors (such as leptin and TNF-α). Among the components found in the extract of *Toddalia asiatica*, aculeatin was found to promote the differentiation of mouse 3T3-L1 preadipocytes into adipocytes [132]. DMA revealed the involvement of PPAR-γ target genes in the process of activation by aculeatin, which is not a ligand of PPAR-γ, suggesting the presence of additional signaling mechanisms.

Atherosclerosis is a chronic inflammatory response of white blood cells in arterial blood vessels, which is promoted by low-density lipoproteins (LDLs), carriers of cholesterol, and triglycerides, and results in the formation of atherosclerotic plaques that are rich in macrophages and foam cells. Estrogenic activity was detected by DMA-based gene expression profiling in the extract of *Agaricus blazei*, which was attributable to brefeldin A [138]. The extract has no estrogen receptor-dependent cell proliferation activity, while showing activation of estrogen signaling (such as activation of ERK, Akt and P70S6K) and beneficial effects for patients with high levels of oxidized LDLs (see Section 3.3).

Carcinogenesis, alternatively referred to as oncogenesis or tumorigenesis, is a process by which normal cells are transformed into cancer cells characterized by uncontrolled cell division; it involves a progression of changes at the cellular, genetic, and epigenetic levels. Several chemicals exhibiting anti-carcinogenic effects were isolated or identified from natural products, such as 3,3′-diindolylmethane from cruciferous vegetables [145], grifolin from *Albatrellus confluens* [153], paeoniflorin from *Paeonia lactiflora* [161], and quercetin from various dietary plants [161], and further analyzed by DMA. 3,3′-Diindolylmethane is estrogenic and shows gene expression profiles favoring tumor promotion [145]. Grifolin acts negatively against the cell cycle and cell growth through inhibiting ERK and Rb pathways, and downregulates the expression of *cyclin D1*, *cyclin E*, and *CDK4* (a gene for a cyclin-dependent kinase), and upregulates the expression of *CKI* (a CDK inhibitor gene) [153]. Paeoniflorin enhances the expression of HSP70, which helps to protect cells from stress, and modulates the expression of *CDC2*, *FOSL1*, and *EGR1*, regulators of cell growth and proliferation [161]. Quercetin, on the other hand, induces p53-independent apoptosis by enhancing the expression of death-receptor or TNFR signaling genes, such as the genes for caspase-10, DFF45, FAS, IκBα, IL1R (Interleukin-1 receptor), TNFR1, and TRAILR [171].

Inflammation is a protective response to cell injury, and involves the local vascular system, the immune system, and various cells within the injured tissue. Ergosterol peroxide produced by *Sarcodon aspratus* suppresses inflammatory response in macrophages by inhibiting TNF-α secretion and down-regulating the expression of interleukin1α/β (IL-1α/β) through pathways such as C/EBPβ, ERK, JNK, MAPK, and NF-κB [147].

Apoptosis is the process of programmed cell death that may occur in multicellular organisms in response to various stresses, such as heat, hypoxia, increased intracellular calcium concentration, nutrient deprivation, receptor–ligand binding, radiation, and viral infection. Several chemicals are related to the promotion of apoptosis and thus have been used as effective components in herbal medicine. Emodin extracted from the rhizomes of *Rheum palmatum* showed testicular toxicity, including the induction of apoptosis, most likely through pathways such as IGF-1, TGF/Wnt, and TNFR1 signaling [146]. β-Hydroxyisovalerylshikonin extracted from *Lithospermum erythrorhizon* is an inhibitor of protein-tyrosine kinases and induces apoptosis by suppressing TRAP1, a TNF-associated protein and a member of the HSPs, as well as the production of ROS [155]. Tanshinone IIA found in the root of *Salvia miltiorrhiza* induces peroxisome proliferator-activated receptor (PXR)/NF-κB/CCL2-mediated apoptosis in leukemia cells [180]. 2,4,3′,5′-Tetramethoxystilbene extracted from fruit, berries, and grapes is a derivative of resveratrol and a strong inducer of apoptosis by increasing the expression of tubulin, stress response, and pro-apoptotic genes [178].

Chemoprevention refers to the administration of a medication, such as drugs and vitamins, for the purpose of preventing disease or infection, and various chemicals have been developed especially for cancer chemoprevention. Myricetin [158] and sulforaphane [177] isolated from dietary plants show chemopreventive activity against cancer through activating Nrf2-mediated antioxidant response or PI3K/Akt signaling pathways, respectively.

Hypoxia is a condition in which a cell is deprived of adequate oxygen supply and has been shown to stimulate various biological and physiological responses. Paeonol isolated from *Paeonia suffruticosa* induces the expression of hypoxia-inducible genes, including hypoxia-inducible factor 1 (HIF-1)-target genes, through suppressing the NF-κB signaling pathway and inhibiting amyloid precursor protein (APP) activity [162].

Life extension has been studied in terms of slowing down or reversing the processes of aging in order to extend both the maximum and the average lifespan, and the effects of anti-aging products, nutrition, physical fitness, skin care, hormone replacements, vitamins, supplements, and herbs have been examined. Diallyl trisulfide isolated from garlic increases the longevity of nematodes through activation of the pro-longevity transcription factor gene *skn-1* and the products of its target genes [144].

Conditions such as chronic (arthritis, asthma, cancer, diabetes, and viral diseases) and neurodegenerative (Parkinson’s and Alzheimer’s diseases) diseases have been treated with TCM [1], among which some were investigated by DMA and explored by animal tests and/or clinical studies to eventually achieve clinical applications. Other than the cell functions discussed above, the diseases with extensive impacts were also investigated. For example, antidepressant, anti-diabetic, anti-obesity, neuromodulation, and neuroprotection effects, and the treatments of neurological, Parkinson’s, and Alzheimer’s diseases associated with TMC and/or constituent herbs/mushrooms/dietary plants were studied by means of DMA (Table 1), or their effective components, such as ginsenosides (for diabetes), (−)-hydroxycitric acid (for obesity), obovatol (for neuroinflammation), and salvianolic acid B (for neuroprotection), were studied by means of DMA (Table 2).

### 3.3. Activities Found by DNA Microarray Assays (Silent Estrogens)

Activities found by DMA are often detected as cell signals in specific pathways, such as angiogenesis, ErbB/HER, MAPK, nuclear receptor, and ubiquitin/proteasome signaling pathways, and/or in cell functions, such as apoptosis, autophagy, cell cycle/DNA damage/cytoskeletal formation, cellular metabolism, chromatin/epigenesis regulation, development/differentiation, immunology/inflammation response, neurological diseases, and translational control [183]. While most of these cell signaling pathways and cell functions can be detected by other technologies, there might be some activities that can be detected exclusively by DMA. One such activity is by a group of estrogens, silent estrogens, which show estrogenic gene expression profiles without showing positive effects on cell proliferation [13].

Estrogen is a female hormone that is responsible for various biological and physiological activities, including receptor-mediated stimulation of the proliferation of cells in tissues such as the breast and ovary. Several chemicals and mixtures of chemicals, such as brefeldin A [138], licorice extracts [150], and oil degradation products [184], were found to show gene expression profiles similar to that for estrogen, although they did not stimulate the proliferation of estrogen receptor-positive breast cancer MCF-7 cells. Although the signaling pathway for cell proliferation could theoretically be separated from those for other cell functions, this separation has not been possible because most of the cells examined for estrogenic activity contain estrogen receptors and the technologies used were not suited to such a purpose. Recent findings about more complicated signaling pathways/networks, such as autocrine/paracrine/homeostatic networks and crosstalk/bypassing of cell signals, include pathways not necessarily involving cell proliferation or the cells containing estrogen receptors [185,186]. Thus, estrogenic activity can be detected even for silent estrogens because DMA can separate various signaling pathways, and the similarity of chemicals can be analyzed at the levels of gene expression and cell signaling.

## 4. Applications of DNA Microarray Assays for Traditional Chinese Medicine

Modernization of TCM has been discussed in association with several key issues, such as the material basis of TCM formulas, the quality evaluation system and evaluation of the efficiency and safety of TCM formulas; key technological tools in systems biology, such as those in genomics, interactomics, metabonomics, phenomics, and proteomics, could also be used to understand the chemome of TCM, an integrated world of the external TCM system and the internal human body [2]. TCMs, such as Beimu (*Fritillaria* spp.), Chishao (*Paeonia* spp.), Chuanxiong (*Ligusticum chuanxiong*), Chuipencao (*Sedum sarmentosum*), Danggui (*Angelica sinensis*), Danshen (*Salvia miltiorrhiza*), Dongchongxiacao (*Cordyceps sinensis*), Ezhu/Yujin (rhizome and radix of *Curcuma*), Guanghuoxiang (*Pogostemon cablin*), Huangqi (*Astragalus* spp.), Jinyinhua (*Lonicera japonica*), Juhua (*Chrysanthemum morifolium*), Lingzhi (*Ganoderma* spp.), Sanqi (*Panax notoginseng*), Wuweizi (*Schisandra chinensis*), and Yingyanghuo (*Epimedium* spp.), were analyzed by biophysical techniques such as capillary electrophoresis, gas chromatography, HPLC, mass spectrometry (MS), thin-layer chromatography (TLC), ultra-performance liquid chromatography (UPLC), and ultraviolet/near-infrared spectrometry, by molecular biological techniques such as genomic PCR and RT-PCR, or by immunological assays such as enzyme-linked immunosorbent assay (ELISA) for screening and/or quality control of effective components, which include alkaloids, cyanophoric glycosides, ergosterol, essential oils, flavonoids, iridoid glycosides, lignans, paeoniflorin, phenolic acids, saponins, steroids, sugars/polysaccharides, and triterpenoids [5].

Chinese medicinal plants were analyzed by DNA-based technologies, such as those detecting amplified fragment length polymorphism (AFLP), cleaved amplified polymorphic sequence (CAPS), inter-simple sequence repeat (ISSR), random amplified polymorphic DNA (RAPD), restriction fragment length polymorphism (RFLP), and simple sequence repeat (SSR), and those by amplification refractory mutation system (ARMS), DNA amplification fingerprinting (DAF), hybridization, microarray assay and sequencing [8], or by assays at the cell/tissue/animal levels, such as gene knockout and cell membrane chromatography and transgenics [187].

The literature surveyed for transcriptomics using DMA in the study of 297 frequently used medicinal herbs in China showed that most of the studies focused on finding their biological effects [10]. However, there are cases where DMA was applied to screen effective components and for quality control of TCM. In this section, we discuss how DMA has been used for quality control of TCM.

Additional applications of effective chemicals in TCM analyzed by DMA include patent filing, drug discovery, and clinical trials. An effective chemical identified and characterized by these applications can be patented as a new chemical, or otherwise as a known chemical with a new application.

### 4.1. DNA Microarray Assays for Quality Control of Traditional Chinese Medicine

DMA has been used to detect and evaluate various activities of pure chemicals and mixtures of chemicals [13,188]. When DMA was applied for the study of TCM, gene sets specific to herbs were selected. For example, a set of 55 genes were screened by DMA in order to understand the effect of Qingfei Xiaoyan Wan formula on asthma by the regulation of gene/protein networks [80]. A set of 92 genes was found to be differentially regulated by Toki-shakuyaku-san, a formula effective for circulation problems [89]. A set of nine marker genes was initially screened by DMA and subsequently confirmed by RT-PCR to assess the batch-to-batch consistency of the biological effects of ISF-1, a formula used for the management of post-stroke disorders [6]. 

Once gene sets have been selected, they can be used for screening and/or quality control of the herbs. For example, DMA was used to screen TCM species with inhibitory effects on Cytochrome P450 (CYP450) intended to treat HIV infection [189]. High-throughput DMA were applied to screen for anti-mitotic effects (independent of toxicity) on the proliferation of MDA-MB-231 cells from 897 aqueous extracts of commonly used natural products, and less than 1.34% of the extracts tested showed growth inhibitory properties at a concentration of less than 0.0183 mg/mL [27]. The DMA based on the yeast transcriptome was used for quality control of the extracts of *Equisetum arvense* [30].

Specific activity was examined to evaluate the quality of materials in food and supplements. For example, estrogenic activity was examined by DMA using a customized DNA microarray containing 172 estrogen-responsive genes in order to evaluate food materials, such as phytoestrogens [127] and ginsenosides [148], and for the extracts of plants and mushrooms, such as soybeans [127], *Glycyrrhiza glabra* [150], and *Agaricus blazei* [138]. The estrogen-responsive genes were further classified into six functional groups (enzymes, signaling, proliferation, transcription, transport, and others) and some showed preferences for specific groups [183].

### 4.2. Protocols of DNA Microarray Assays for Quality Control

**Figure 2:**
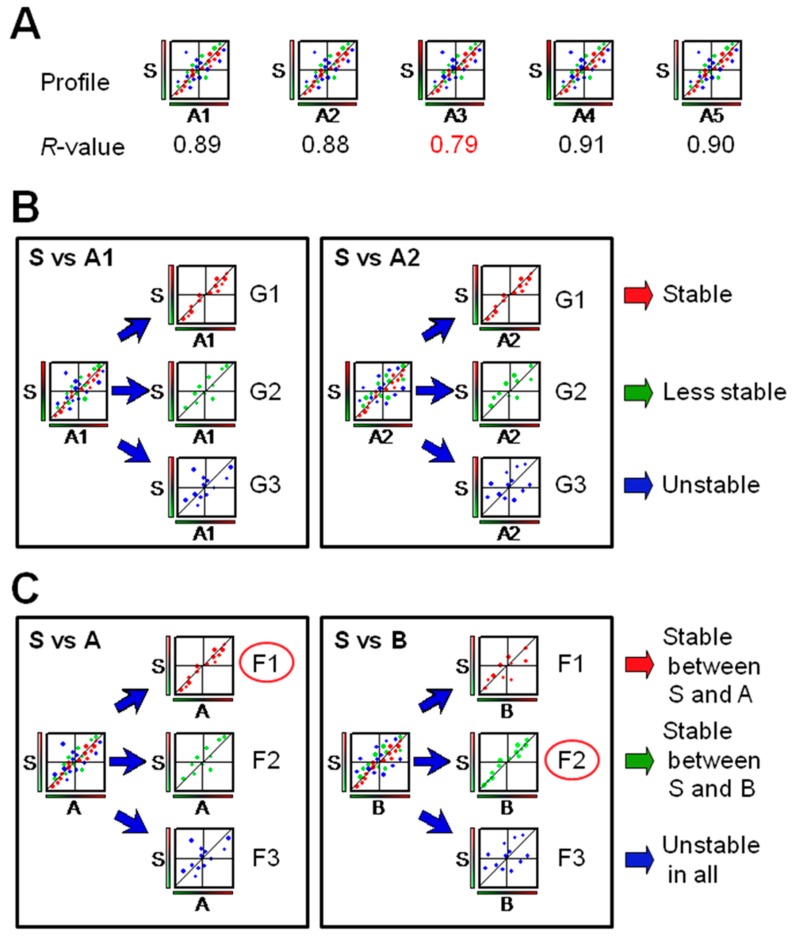
Quality control of herbal components by gene expression profiling. Examples of application of DMA for quality control of TCM are summarized. (**A**) A simplified protocol of quality control of herbal extracts. Gene expression profiles for different sources of herbal extracts (A1 to A5) are compared with that of a standard (S) using correlation coefficients (*R*-values) based on linear regression. A case of deviation (A3) can be detected by comparing *R*-values for the profiles of the genes appropriately selected. (**B**) Selection of gene sets for gene expression profiling-based quality control. The degree of stability in quality control can be influenced and controlled by selecting arbitrarily grouped genes (G1 to G3), which show stable (G1), less stable (G2) or unstable (G3) reproducibility upon comparing various lots (A1 and A2) of the herbal extracts with a standard (S). (**C**) Selection of gene sets for gene function-based quality control. The profiling shown in (**B**) can be performed with functionally grouped genes (F1 to F3) to give a protocol of efficacy-based quality control of herbal extracts.

Schemes of new DMA-based protocols for quality control of herbal extracts are summarized in Figure 2. A simplified protocol is based on gene expression profiles of different sources of herbal extracts (A1 to A5), which are compared with that of a standard (S) by correlation coefficients (*R*-values) based on linear regression (Figure 2A). Deviations (such as in A3, Figure 2A) can be detected by comparing *R*-values. While gene sets for expression profiling can be used without selection (Figure 2A), the genes having specific cell functions could be used to improve the level of quality control (Figure 2B,C), where the gene sets (G1 to G3) can be selected arbitrarily, such as those showing stable (G1), less stable (G2) or unstable (G3) reproducibility when various lots (A1 and A2) of the herbal extracts are compared with a standard (Figure 2B). Alternatively, gene sets can be selected according to gene functions (Figure 2C), where profiling can be performed with functionally grouped genes (F1 to F3) to give a protocol of efficacy-based quality control of herbal extracts. The advantages for using these protocols are that: (1) cell function-based gene expression profiling is good for efficacy-based quality control; (2) gene sets can be selected depending on the purpose; and (3) various activities can be monitored by different gene sets.

## 5. Conclusions and Perspectives

Owing to the progress of new biotechnological tools, new approaches have been developed, including systems biology, signal transduction study, and RNA-sequencing-based whole genome/exome analysis, which provide us with massive amounts of omics data. However, we still do not know how to use these tools effectively. For example, we now know a number of chemicals showing estrogenic activity and the number is steadily increasing because they affect cells, tissues, and organs through complex and novel pathways of cell signaling, such as intracellular signal transduction, signal crosstalk/bypassing, and intercellular networks of autocrine/paracrine signaling [185,186]. This complex status would be the same as in the study and the application of TCM because, since estrogenic activity is one of the most important activities of effective components in herbs and has been shown to affect the human body, the findings about estrogen would be true for more complex statuses, which include other hormones and growth factors. Chemicals having other hormonal and growth-factor activities could show such complexities, and thus new approaches are needed to understand the effects of chemicals. Other than the pharmacological use of chemicals, DMA-based gene expression profiling and pathway-based testing of chemicals have been developed for the diagnosis of diseases and risk assessment of endocrine disruptors, where the development of new types of diagnostic tool is in progress, such as the in vitro diagnostic multivariate index assay (IVDMIA), and toxicity pathway-based risk management protocols, such as those proposed by the U.S. National Research Council [190]. Thus, a pathway-based evaluation of beneficial effects and the assessment of potential risks by means of omics technologies are needed for the study and development of TCM.

## Figures and Tables

**Table 1:** Herbs, traditional medicines, mushrooms, and dietary plants analyzed by DNA microarray assay (DMA).

Source	Extract or Material Examined	Pathway or Function Examined/Identified	Reference (Assay ^a^)
**Herb**			
*Actaea racemosa* (Black cohosh)	Extract	Anti-carcinogenesis	Einbond et al., 2012 [14] (A, C, T)
*Angelica sinensis* (Dong quai)	Extract	Wound healing	Zhao et al., 2012 [15] (C, T)
*Anoectochilus formosanus* (an orchid)	Extract	Anti-carcinogenesis	Yang et al., 2004 [16] (C, T)
*Astragalus propinquus*, *Rehmannia glutinosa*	Radix extract	Wnt signaling/Angiogenesis	Zhang et al., 2011 [17] (C, P, T)
*Boswellia serrata* (Salai)	Extract	Anti-inflammation	Kiela et al., 2005 [18] (A, C, R, T)
*Chelidonium majus* (Greater celandine)	Extract (Alkaloids)	Anti-carcinogenesis	El-Readi et al., 2013 [19] (C, T)
*Chrysanthemum lavandulifolium* (Mum)	Extract	Antibiotic	Kim et al., 2013 [20] (T)
*Cistanche tubulosa*	Root extract	Anti-atherosclerosis	Shimoda et al., 2009 [21] (A, T)
*Coptis chinensis* (Chinese goldthread)	Rhizome extract	p53 signaling	Cheng et al., 2008 [22] (C, T)
*Coptis japonica* (Goldthread)	Rhizome extract	Anti-carcinogenesis	Iizuka et al., 2003 [23] (C, T)
*Coptis japonica* (Goldthread)	Rhizome extract	IFβ/TNF-α/Apoptosis	Kang et al., 2005 [24] (C, P, T)
*Curcuma longa* (Turmeric)	Extract	Anti-inflammation	Kim et al., 2013 [25] (A, C, T)
*Curcuma longa* (Turmeric)	Essential oil	Anti-diabetic effect	Honda et al., 2006 [26] (A, T)
*Dioscorea villosa* (Wild yam), *Lithospermum canescens* (Alkanet root), *Trillium erectum* (Beth root)	Extract	Anti-mitotic effect	Mazzio et al., 2014 [27] (C, T)
*Echinacea purpurea* (Purple coneflower)	Extract	Immune response	Wang et al., 2006 [28] (C, T)
*Echinacea purpurea* (Purple coneflower)	Extract	Immune response	Wang et al., 2008 [29] (C, P, T)
*Equisetum arvense* (Field horsetail)	Extract	Metabolism/Stress response	Cook et al., 2013 [30] (C, T)
*Eupatorium perfoliatum* (Boneset)	Extract	Anti-inflammation	Maas et al., 2011 [31] (C, P, T)
*Hedyotis diffusa*	Extract	Anti-carcinogenesis	Kuo et al., 2015 [32] (C, T)
*Hypericum perforatum* (St. John’s wort)	Extract	Antidepressant	Wong et al., 2004 [33] (A, T)
*Hypericum perforatum* (St. John’s wort)	Extract	Neurological disease/Angiogenesis	McCue & Phang, 2008 [34] (A, P, T)
*Levisticum officinale* (Lovage)	Essential oil	Cell proliferation	Sertel et al., 2011 [35] (C, T)
*Nelumbo nucifera* (Lotus)	Seed extract	MAPK/NO/Anti-inflammation	Sohn et al., 2009 [36] (C, P, T)
*Nelumbo nucifera* (Lotus)	Seed extract	Neuroprotection	Lee et al., 2010 [37] (A, T)
*Paeonia lactiflora* (Chinese peony)	Root extract	Apoptosis	Lee et al., 2002 [38] (C, T)
*Paeonia suffruticosa* (Moutan)	Extract	Anti-inflammation	Yun et al., 2012 [39] (C, T)
*Panax quinquefolius* (American ginseng)	Extract	Anti-carcinogenesis	Luo et al., 2008 [40] (C, T)
*Piper methysticum* (Kava)	Extract	Hepatotoxicity	Guo et al., 2010 [41] (A, T)
*Piper methysticum* (Kava)	Extract	Hepatotoxicity	Guo et al., 2009 [42] (A, T)
*Salvia miltiorrhiza*, *Pueraria lobata*	Root extract	MAPK/Insulin signaling	Fong et al., 2011 [43] (C, P, T)
*Scrophularia ningpoensis* (Chinese figwort)	Extract	MAPK/NF-κB/Apoptosis	Shen et al., 2012 [44] (C, P, T)
*Scutellaria barbata* (Barbed skullcap)	Extract	Anti-carcinogenesis	Yin et al., 2004 [45] (C, T)
*Sutherlandia frutescens* (Cancer bush)	Extract	Apoptosis	Stander et al., 2007 [46] (C, T)
*Tabebuia avellandae* (Pink Lapacho)	Extract	Apoptosis	Mukherjee et al., 2009 [47] (C, T)
*Tripterygium wilfordii* (Leigongteng)	Extract	PPAR/Hepatotoxicity	Zhang et al., 2012 [48] (A, T)
*Vitex rotundifolia* (Beach vitex)	Extract	MAPK/Anti-inflammation	Sohn et al., 2009 [49] (C, T)
**Mushroom**			
*Agaricus bisporus* (Common mushroom)	Extract	Anti-carcinogenesis	Adams et al., 2008 [50] (C, T)
*Agaricus blazei* (Himematsutake)	Extract	Immune response	Ellertsen et al., 2006 [51] (P, T)
*Agaricus blazei*	Extract	Anti-carcinogenesis	Grinde et al., 2006 [52] (C, T)
*Agaricus blazei*	Mycelia extract	ERK/Anti-atherosclerosis	Dong et al., 2012 [53] (C, P, T)
*Cordyceps sinensis* (Caterpillar fungus)	Extract	TLR signaling	Li et al., 2012 [54] (C, T)
*Daedalea gibbosa* (Lumpy bracket)	Extract	NF-κB/NO production	Ruimi et al., 2010 [55] (C, R, T)
*Ganoderma lucidum* (Lingzhi)	Extract (Polysaccharide-rich)	Apoptosis	Cheng et al., 2007 [56] (C, P, T)
*Ganoderma lucidum* (Lingzhi)	Extract	Anti-metastatic effect	Loganathan et al., 2014 [57] (A, C, T)
*Ganoderma sinense* (Lingzhi)	Extract	NF-κB/Anti-inflammation	Cheng et al., 2010 [58] (T)
*Grifola frondosa* (Maitake)	Extract	Anti-arteriosclerosis	Sato et al., 2013 [59] (A, T)
*Grifola frondosa* (Maitake), *Hypsizigus marmoreus* (Buna-shimeji)	Extract	TLR3/IF/Immune response	Sato et al., 2011 [60] (A, T)
*Lentinus edodes* (Shiitake)	Mycelia extract	(Lignin-rich) Immune response	Kawano et al., 2010 [61] (C, P, T)
Mushroom blend (*Agaricus blazei*, *Cordyceps sinensis*, *Coriolus versicolor*, *Ganoderma lucidum*, *Grifola frondosa*, *Polyporus umbellatus*)	Mycelia extract	Anti-carcinogenesis	Jiang & Sliva, 2010 [62] (C, P, T)
*Pleurotus ostreatus* (Hiratake)	Extract	p53/Apoptosis	Jedinak & Sliva, 2008 [63] (C, P, T)
*Pleurotus ostreatus* (Hiratake), *Ganoderma lucidum, Poria cocos* (Hoelen)	Extract (Triterpene-rich)	Anti-carcinogenesis	Cheng et al., 2013 [64] (C, P, T)
*Trametes versicolor* (Turkey tail)	Extract	Apoptosis	Hsieh & Wu, 2006 [65] (C, P, T)
**TCM/TKM/Kampo**			
Boiogito, Bofutsushosan, Orengedokuto (Kampo)	Mixtures of herbs	Anti-adipogenesis	Yamakawa et al., 2008 [66] (C, T)
Chunggan (TKM)	Mixture of 13 herbs	Anti-fibrotic effect	Kim et al., 2013 [67] (C, P, T)
Danggui Buxue Tang (TCM)	Mixture of two herbs	Proliferation/differentiation	Choi et al., 2011 [68] (C, P, R, T)
Guanxin No.2 decoction (TCM)	Mixture of five herbs	Cardioprotection	Zeng et al., 2009 [69] (A, T)
Hochu-ekki-to (Kampo)	Mixture of 10 herbs	Antidepressant	Tohda et al., 2008 [70] (C, T)
Hochu-ekki-to (Kampo)	Mixture of 10 herbs	Immune response	Matsumoto et al., 2010 [71] (A, P, T)
Huang-Lian-Jie-Du decoction (TCM)	Mixture of four herbs	Alzheimer’s disease	Zheng et al., 2008 [72] (A, T)
Inchin-ko-to (Kampo)	Mixture of three herbs	MAPK/Apoptosis	Sakaida et al., 2003 [73] (A, P, T)
ISF-1 (TCM)	Mixture of seven herbs	Neuroprotection	Rong et al., 2007 [6] (C, T)
Juzen-taiho-to (Kampo)	Mixture of 10 herbs	MAPK/Anti-carcinogenesis	Zheng et al., 2014 [74] (A, T)
Juzen-taiho-to (Kampo)	Mixture of 10 herbs	ISGF3-IRF7/IFα signaling	Munakata et al., 2012 [75] (A, P, T)
Kangxianling (TCM)	Mixture five herbs	TGF-β1/Smad signaling	Dong et al., 2012 [76] (A, P, T)
Kososan (Kampo)	Mixture of rive herbs	Antidepressant	Hayasaki et al., 2007 [77] (C, T)
PC-SPES (TCM)	Mixture of eight herbs	Anti-carcinogenesis	Bonham et al., 2002 [78] (C, T)
Pulsatillae Decoction (TCM)	Mixture of four herbs	Anti-endotoxin action	Hu et al., 2009 [79] (C, T)
Qingfei Xiaoyan Wan (TCM)	Mixture of eight herbs	Anti-inflammation/Anti-remodeling	Zhao et al., 2013 [80] (A, P, T)
Qinggan Huoxuefang (TCM)	Mixture of five herbs	Apoptosis	Ji et al., 2006 [81] (A, T)
Saireito (Kampo)	Mixture 12 herbs	Immune response	Watanabe et al., 2010 [82] (A, T)
S/B remedy	Mixture of two herbs	Cell proliferation	Wang et al., 2005 [83] (A, C, T)
SH21B (TKM)	Mixture of seven herbs	Wnt signaling/Adipogenesis	Lee et al., 2011 [84] (C, P, T)
Si-Wu-Tang (TCM)	Mixture of four herbs	Chemoprevention	Wen et al., 2011 [85] (C, R, T)
Si-Wu-Tang (TCM)	Mixture of four herbs	Gynecological diseases	Fang et al., 2013 [86] (C, T)
TCM	Mixture of three herbs	Estrogen/Anti-osteoporosis	Sun et al., 2008 [87] (A, T)
TCM (15 formulae)	Mixtures of herbs	Anti-carcinogenesis, etc.	Cheng et al., 2010 [88] (A, T)
Toki-shakuyaku-san (Kampo)	Mixture of six herbs	Circulation disorders	Kawamura et al., 2007 [89] (C, T)
Toki-to (Kampo)	Mixture of 10 herbs	Parkinson’s disease	Sakai et al., 2007 [90] (A, T)
VI-28 (TCM)	Mixture five herbs	IGF-1/Immune response	Pan-Hammarström et al., 2006 [91] (C, T)
Xiaoqinglong decoction (TCM)	Mixture of eight herbs	Obstructive lung disease	Zhang et al., 2012 [92] (A, T)
Xuefu Zhuyu decoction (TCM)	Mixture of 11 herbs	Angiogenesis modulation	Song et al., 2012 [93] (C, T)
Youkongdan (TKM)	Mixture 17 herbs	Neuromodulation	Shin et al., 2004 [94] (A, T)
Zeng Sheng Ping (TCM)	Mixture of six herbs	Chemoprevention	Zhang et al., 2004 [95] (A, T)
**Dietary plant (Vegetable, Fruit, and Cereal)**			
*Aframomum angustifolium*	Seed extract	Skin aging prevention	Bonnet-Duquennoy et al., 2007 [96] (C, T)
*Allium sativum* (Garlic)	Extract	Caspase-3/Apoptosis	Su et al., 2006 [97] (C, P, T)
*Allium sativum* (Garlic)	Extract	Anti-carcinogenesis	Frantz et al., 2000 [98] (C, T)
Apple	Extract	NF-κB/Anti-inflammation	Jung et al., 2009 [99] (C, P, T)
*Artemisia dracunculus* (Tarragon)	Extract	Insulin receptor signaling	Wang et al., 2011 [100] (A, T)
Black raspberry	Extract	Anti-carcinogenesis	Wang et al., 2011 [101] (A, T)
Blueberry	Powder	Wnt signaling/Anti-carcinogenesis	Adams et al., 2011 [102] (A, C, P, T)
Broccoli	Extract	TGF-β/Polyamine catabolism	Furniss et al., 2008 [103] (C, P, T)
*Camellia sinensis* (Green tea)	Extract	Lipid metabolism disorder	Suzuki et al., 2013 [104] (A, T)
*Ceratonia silique* (Carob)	Extract (Gallic acid-rich)	Chemoprevention	Klenow et al., 2009 [105] (C, T)
Chungkookjang (Fermented soybean)	Extract	TGF-β/Anti-inflammation	Hwang et al., 2011 [106] (C, T)
Citrus/Grape/Green tea	Extract	Leukocyte function	Salas et al., 2009 [107] (A, T)
*Elaeis guineensis* (Oil palm)	Extract (Phenolics-rich)	Anti-inflammation	Leow et al., 2013 [108] (A, T)
*Fagopyrum esculentum* (Buckwheat)	Sprout extract	Anti-inflammation	Ishii et al., 2008 [109] (A, C, P, T)
*Ginkgo biloba* (Ginkgo)	Extract	Neuromodulation	Watanabe et al., 2001 [110] (A, T)
*Ginkgo biloba* (Ginkgo)	Extract	Anti-carcinogenesis	Rimbach et al., 2003 [111] (T)
Grape	Extract (Oleanolic acid-rich)	Anti-obesity	Yunoki et al., 2008 [112] (A, T)
Grape	Extract (Anthocyanin-rich)	Anti-inflammation	Lefevre et al., 2008 [113] (A, T)
Grape	Seed extract (Proanthocyanidin-rich)	Cardioprotection	Bagchi et al., 2003 [114] (C, T)
Grapefruit	Extract	AhR/Chemoprevention	de Waad et al., 2008 [115] (C, R, T)
Green tea	Extract	Cytotoxicity	Yang et al., 2006 [116] (C, T)
Kiwifruit	Extract	Immune response	Edmunds et al., 2012 [117] (A, P, T)
*Litchi chinensis* (Lychee)	Pericarp extract	Estrogen/Anti-carcinogenesis	Wang et al., 2006 [118] (C, P, T)
*Lithospermum erythrorhizon* (Gromwell)	Extract	Stress response	Bang et al., 2014 [119] (C, T)
*Malus domestica* (Marie Ménard apple)	Powder (Polyphenol-rich)	Anti-inflammation	Castagnini et al., 2009 [120] (A, T)
*Momordica charantia* (Bitter gourd)	Extract	TNF-α/Anti-inflammation	Kobori et al., 2008 [121] (C, P, T)
Nectarine, Peach	Extract	DNA damage prevention	Croteau et al., 2010 [122] (A, P, T)
Olive	Virgin olive oil	Cardioprotection	Camargo et al., 2010 [123] (C, T)
Persimmon	Peel extract	Insulin signaling	Izuchi et al., 2011 [124] (A, P, T)
Pistachio	Oil extract	Inflammatory response	Zhang et al., 2010 [125] (C, T)
*Salacia reticulata* (Kothala himbutu)	Extract	Inflammatory response	Im et al., 2008 [126] (A, T)
Soybean	Extract	Estrogen signaling	Ise et al., 2005 [127] (C, T)
Sweet corn	Powder	Wnt signaling/Anti-carcinogenesis	Tokuji et al., 2009 [128] (A, T)
*Syzygium aromaticum* (Clove)	Extract	Anti-diabetic	Prasad et al., 2005 [129] (C, T)
*Toona sinensis* (Chinese mahogany)	Leaf extract	Apoptosis	Chia et al., 2010 [130] (C, T)
*Vaccinium myrtillus* (Bilberry)	Powder	MAPK/Vision	Mykkänen et al., 2012 [131] (A, T)

^a^ Abbreviations for assays are: animal test (A), cell-proliferation assay (C), protein assay such as Western blotting and immunoassay (P), reporter gene assay (R) and transcription assay (such as RT-PCR and DNA microarray assay) (T). AhR: aryl hydrocarbon receptor; ERK: extracellular signal-regulated kinase; IF: interferon; IGF-1: insulin-like growth factor 1; IRF7: Interferon regulatory factor 7; ISGF3: Interferon-stimulated gene factor 3; MAPK: mitogen-activated protein kinase; NF-κB: nuclear factor κ-light-chain-enhancer of activated B cells; NO: nitric oxide; PPAR: peroxisome proliferator-activated receptor; TCM: traditional Chinese medicine; TGF: tumor growth factor; TKM: traditional Korean medicine; TLR: Toll-like receptor; TNF: tumor necrosis factor.

**Table 2:** Effective chemicals analyzed by DMA.

Chemical Examined (Category)	Major Source Examined/Identified	Pathway or Function	Reference (Assay ^a^)
Actein (Terpenoid)	*Actaea racemosa* (Black cohosh)	Anti-carcinogenesis	Einbond et al., 2012 [14] (Table 1)
Aculeatin (Coumarin)	*Toddalia asiatica* (Orange climber)	PPAR-γ/Adipogenesis	Watanabe et al., 2014 [132] (C, T)
Baicalin/Deoxycholic acid/Jasminoidin	Qing-Kai-Ling (TCM)	Ischemic stroke	Li et al., 2012 [133] (A, T)
Berberine (Alkaloid)	*Coptis japonica* (Goldthread)	Anti-carcinogenesis	Iizuka et al., 2003 [23] (Table 1)
Berberine (Alkaloid)	*Coptis chinensis* (Chinese goldthread)	Anti-infectious	Zhang et al., 2009 [134] (T)
Berberine, etc.	*Coptis japonica* (Goldthread)	Anti-carcinogenesis	Hara et al., 2005 [135] (T)
Biochanin A/Genistein (Flavonoid)	Plant (fruit/vegetables/leaves/grains)	Anti-carcinogenesis	Moon et al., 2007 [136] (C, T)
Boswellic acid (Terpenoid)	*Boswellia serrata* (Salai)	Chemoprevention	Shen et al., 2012 [137] (C, T)
Brefeldin A (Lactone)	*Agaricus blazei*	ERK/Anti-atherosclerosis	Dong et al., 2013 [138] (C, P, T)
Celastrol (Terpenoid)	*Tripterygium wilfordii* (Leigongteng)	Anti-carcinogenesis	Pham et al., 2010 [139] (C, P, T)
Celastrol (Terpenoid)	*Celastrus scandens* (Bittersweet)	Immune response	Yu et al., 2012 [140] (T)
Chelidonine (Alkaloid)	*Chelidonium majus* (Greater celandine)	Anti-carcinogenesis	El-Readi et al., 2013 [19] (Table 1)
Curcumin (Diarylheptanoid)	*Curcuma longa* (Turmeric)	Apoptosis	Ramachandran et al., 2005 [141] (C, T)
Curcumin (Diarylheptanoid)	*Curcuma longa* (Turmeric)	Anti-oxidative response	Meja et al., 2008 [142] (C, P, T)
Curcumin (Diarylheptanoid)	*Curcuma longa* (Turmeric)	Life-span extension	Lee et al., 2010 [143] (T)
Diallyl trisulfide (Organosulfur)	*Allium sativum* (Garlic)	skn-1/Life-span extension	Powolny et al., 2011 [144] (T)
3,3′-Diindolylmethane	Cruciferous vegetable	Estrogen/Carcinogenesis	Tilton et al., 2007 [145] (A, T)
Emodin (Anthraquinone)	*Rheum palmatum* (Turkish rhubarb)	TNFR1/IGF-1R/Apoptosis	Oshida et al., 2011 [146] (T)
Ergosterol peroxide (Steroid)	*Sarcodon aspratus*	NF-κB/Anti-inflammation	Kobori et al., 2007 [147] (C, P, T)
Ginsenosides F1/Rb1/Rg1/Rh1 (Terpenoid)	Ginseng	Estrogen signaling	Dong & Kiyama, 2009 [148] (T)
Ginsenoside Re (Terpenoid)	Ginseng	Anti-diabetic response	Xie et al., 2005 [149] (T)
Ginsenoside Rg3 (Terpenoid)	*Panax quinquefolius* (American ginseng)	Anti-carcinogenesis	Luo et al., 2008 [40] (Table 1)
Glycyrrhizin (Saponin)	*Glycyrrhiza glabra* (Licorice)	Estrogen signaling	Dong et al., 2007 [150] (T)
Glycyrrhizin (Saponin)	*Glycyrrhiza glabra* (Licorice)	Anti-inflammation	Schröfelbauer et al., 2009 [151] (P, T)
Grape antioxidant dietary fiber	Cencibel red grape	Anti-carcinogenesis	Lizarraga et al., 2011 [152] (A, T)
Grifolin (Phenol)	*Albatrellus confluens*	ERK/Anti-carcinogenesis	Ye et al., 2007 [153] (C, P, T)
(−)-Hydroxycitric acid	*Garcinia gummi-gutta* (Garcinia cambogia)	Anti-obesity	Roy et al., 2007 [154] (T)
β-Hydroxyisovalerylshikonin (Quinone)	*Lithospermum erythrorhizon* (Purple gromwell)	ROS/Apoptosis	Masuda et al., 2004 [155] (C, P, T)
Ligustrazine (Tetramethylpyrazine)	*Ligusticum chuangxiong*	Cardioprotection	Li et al., 2004 [156] (T)
Lycopene (Carotenoid pigment)	Tomato	Stress response/Anti-carcinogenesis	Tan et al., 2014 [157] (A, T)
Myricetin (Flavonoid)	Plant (fruits/vegetables/herbs)	Nrf2 /ARE/Chemoprevention	Qin et al., 2013 [158] (T)
Obovatol (Phenol)	*Magnolia obovata* (Japanese bigleaf magnolia)	Neuroinflammation	Ock et al., 2010 [159] (A, C, P, T)
Oil palm phenolics	*Elaeis guineensis* (African oil palm)	Cardioprotection	Leow et al., 2011 [160] (A, T)
Paeoniflorin (Terpenoid)	*Paeonia lactiflora* (Chinese peony)	HSP70/Anti-carcinogenesis	Salunga et al., 2007 [161] (C, P, T)
Paeonol (Phenol)	*Paeonia suffruticosa* (Moutan)	NF-κB/Hypoxia	Su et al., 2010 [162] (P, R, T)
Paeonol/Paeoniflorin/Albiflorin	*Paeonia lactiflora* (Chinese peony)	Anti-inflammation	Huang et al., 2008 [163] (C, T)
PGG (Gallotannin)	*Rhus chinensis* (Sumac)	Anti-carcinogenesis	Yu et al., 2011 [164] (C, P, T)
Phytosterol mixture	Wood (Tall oil)	Anti-atherosclerosis	Xu et al., 2008 [165] (A, T)
Plant phospholipid/lipid conjugate	Plant (nuts/seeds/oils)	Anti-carcinogenesis	Shuman Moss et al., 2014 [166] (A, T)
Plumbagin (Quinone)	*Anoectochilus formosanus* (an orchid)	Anti-carcinogenesis	Yang et al., 2004 [16] (Table 1)
Polysaccharide-K (Krestin)	*Trametes versicolor* (Turkey tail)	Anti-carcinogenesis	Yoshikawa et al., 2004 [167] (C, T)
Polysaccharides	Apple	Anti-carcinogenesis	Li et al., 2012 [168] (C, P, T)
PUFA (*n-3*)	Corn, Olive	Anti-carcinogenesis	Kachroo et al., 2011 [169] (A, T)
PUFAs	*Camelina sativa* (False flax)	Cell proliferation	Morais et al., 2012 [170] (A, T)
Quercetin (Flavonoid)	Plant (fruits/vegetables/leaves/grains)	NF-κB/Anti-carcino- genesis	Youn et al., 2013 [171] (C, P, T)
Resveratrol (Stilbenoid)	Red grape	Vasoprotection	Nicholson et al., 2008 [172] (Review)
Saffron	*Crocus sativus* (Saffron crocus)	Neuroprotection	Natoli et al., 2010 [173] (A, T)
Salvianolic acid B (Phenolic acid)	*Salvia miltiorrhiza* (Red sage)	Anti-carcinogenesis	Yang et al., 2011 [174] (C, P, T)
Sesamin/Episesamin/Sesamolin (Lignan)	Sesame	Lipid metabolism	Ide et al., 2009 [175] (A, T)
Sparstolonin B (Isocoumarin)	*Sparganium stoloniferum* (Bur-reed)	Angiogenesis	Bateman et al., 2013 [176] (C, T)
Sulforaphane (Organosulfur)	Broccoli	PI3K/Akt /Chemoprevention	Melchini et al., 2012 [177] (C, P, T)
2,4,3′,5′-Tetramethoxystilbene (Stilbenoid)	Berry, Grape	Bax/Apoptosis	Aiyar et al., 2010 [178] (T)
Tanshinone IIA (Quinone)	*Salvia miltiorrhiza* (Red sage)	Rho/ROCK/Cell migration	Li et al., 2014 [179] (C, P, T)
Tanshinone IIA (Quinone)	*Salvia miltiorrhiza* (Red sage)	NF-κB/Apoptosis	Liu et al., 2012 [180] (C, P, T)
Triptolide (Terpenoid)	*Tripterygium wilfordii* (Leigongteng)	Immune response, etc.	Chen et al., 2007 [181] (A, T)

^a^ Abbreviations for assays are: animal test (A), cell-proliferation assay (C), protein assay (such as Western blotting and immunoassay) (P), reporter-gene assay (R) and transcription assay (such as RT-PCR and DNA microarray assay) (T). ARE: antioxidant response element; ERK: extracellular signal-regulated kinase; HSP70: 70 kilodalton heat shock protein; IGF-1R: insulin-like growth factor 1 receptor; NF-κB: nuclear factor κ-light-chain-enhancer of activated B cells; PGG: 1,2,3,4,6-Penta-*O*-galloyl-β-d-glucose; PI3K: phosphatidylinositol-3-kinase; PPAR: peroxisome proliferator-activated receptor; PUFA: polyunsaturated fatty acid; ROCK: Rho-associated protein kinase; ROS: reactive oxygen species; TCM: traditional Chinese medicine; TNFR1: tumor necrosis factor receptor 1.
